# Protracted Administration of L-Asparaginase in Maintenance Phase Is the Risk Factor for Hyperglycemia in Older Patients with Pediatric Acute Lymphoblastic Leukemia

**DOI:** 10.1371/journal.pone.0136428

**Published:** 2015-08-28

**Authors:** Hideki Yoshida, Toshihiko Imamura, Akiko M. Saito, Yoshihiro Takahashi, So-ichi Suenobu, Daiichiro Hasegawa, Takao Deguchi, Yoshiko Hashii, Hirohide Kawasaki, Mikiya Endo, Hiroki Hori, Nobuhiro Suzuki, Yoshiyuki Kosaka, Koji Kato, Keiko Yumura-Yagi, Junichi Hara, Megumi Oda, Atsushi Sato, Keizo Horibe, Japan Association of Childhood Leukemia Study (JACLS)

**Affiliations:** 1 Department of Pediatrics, Kyoto Prefectural University of Medicine, Graduate School of Medical Science, Kyoto, Japan; 2 Clinical Research Center, National Hospital Organization Nagoya Medical Center, Nagoya, Japan; 3 Department of Pediatrics, Aomori Prefectural Central Hospital, Aomori, Japan; 4 Division of General Pediatrics and Emergency Medicine, Department of Pediatrics, Oita University, Oita, Japan; 5 Department of Hematology/Oncology, Hyogo Prefectural Children’s Hospital, Kobe, Japan; 6 Department of Pediatrics, Mie University, Tsu, Japan; 7 Department of Pediatrics, Osaka University, Suita, Japan; 8 Department of Pediatrics, Kansai Medical University, Hirakata, Japan; 9 Department of Pediatrics, Iwate Medical University, Morioka, Japan; 10 Department of Pediatrics, Hokkaido Medical Center for Child Health and Rehabilitation, Sapporo, Japan; 11 Department of Hematology Oncology, Children’s Medical Center, Japanese Red Cross Nagoya First Hospital, Nagoya, Japan; 12 Yumura Clinic, Osaka, Japan; 13 Department of Pediatric Hematology/Oncology, Osaka City General Hospital, Osaka, Japan; 14 Department of Pediatrics, Okayama University, Okayama, Japan; 15 Department of Pediatric Hematology/Oncology, Miyagi Children’s Hospital, Sendai, Japan; University of Heidelberg, GERMANY

## Abstract

Although L-asparaginase related hyperglycemia is well known adverse event, it is not studied whether the profile of this adverse event is affected by intensification of L-asparaginase administration. Here, we analyzed the profile of L-asparaginase related hyperglycemia in a 1,176 patients with pediatric acute lymphoblastic leukemia treated according to the Japan Association of Childhood Leukemia Study ALL-02 protocol using protracted L-asparaginase administration in maintenance phase. We determined that a total of 75 L-asparaginase related hyperglycemia events occurred in 69 patients. Although 17 events (17/1176, 1.4%) developed in induction phase, which was lower incidence than those (10–15%) in previous reports, 45 events developed during the maintenance phase with protracted L-asparaginase administration. Multivariate analysis showed that older age at onset (≥10 years) was a sole independent risk factor for L-asparaginase-related hyperglycemia (*P*<0.01), especially in maintenance phase. Contrary to the previous reports, obesity was not associated with L-asparaginase-related hyperglycemia. These findings suggest that protracted administration of L-asparaginase is the risk factor for hyperglycemia when treating adolescent and young adult acute lymphoblastic leukemia patients.

## Introduction

The majority of current pediatric acute lymphoblastic leukemia (ALL) cases have a high long-term survival rate due to the recent advances in treatment using multiple chemotherapeutic agents including L-asparaginase (L-asp) [1]. Since Hill et al first used L-asp in 1967 [2], it has been established as the "key drug" for this disease [3–6]. In addition, a high dose of L-asp is currently used to treat adolescent and young adult (AYA) patients with ALL because the pediatric ALL protocol is superior to the adult protocol for treatment of AYA patients [7, 8]. However, this agent is associated with various complications, including pancreatitis, thrombosis, hypersensitivity, and metabolic disorders [9–11]. Loeb E et al first reported L-asp-induced hyperglycemia in 1970 [12], and impaired glucose tolerance develops in 10% of ALL patients treated with L-asp [13–16]. Severe conditions associated with hyperglycemia, such as diabetic ketoacidosis, may require temporary withdrawal of chemotherapy. Although several studies have determined the obesity, which was surrogate marker for insulin resistance, was the risk factors for L-asp-related hyperglycemia in western countries [15, 16], the profile of adverse events caused by L-asp, especially hyperglycemia may be influenced by ethnicity, schedule/dose of L-asp and concomitant use of steroid [17]. However, these factors have not been studied in large cohort. The purpose of this study was to identify the clinical features and risk factors for L-asp-related hyperglycemia in 1,176 pediatric ALL patients registered in the Japan Association of Childhood Leukemia Study (JACLS) ALL-02 study using protracted L-asp administration in maintenance phase for the patients older than 10 years.

## Methods

### Ethical statement

The written informed consent was obtained from the patients’ guardians according to the Declaration of Helsinki, and the treatment protocols were approved by the institutional review boards of National Hospital Organization Nagoya Medical Center and the participating institutes.

### Patient cohort and treatment

**Fig 1:**
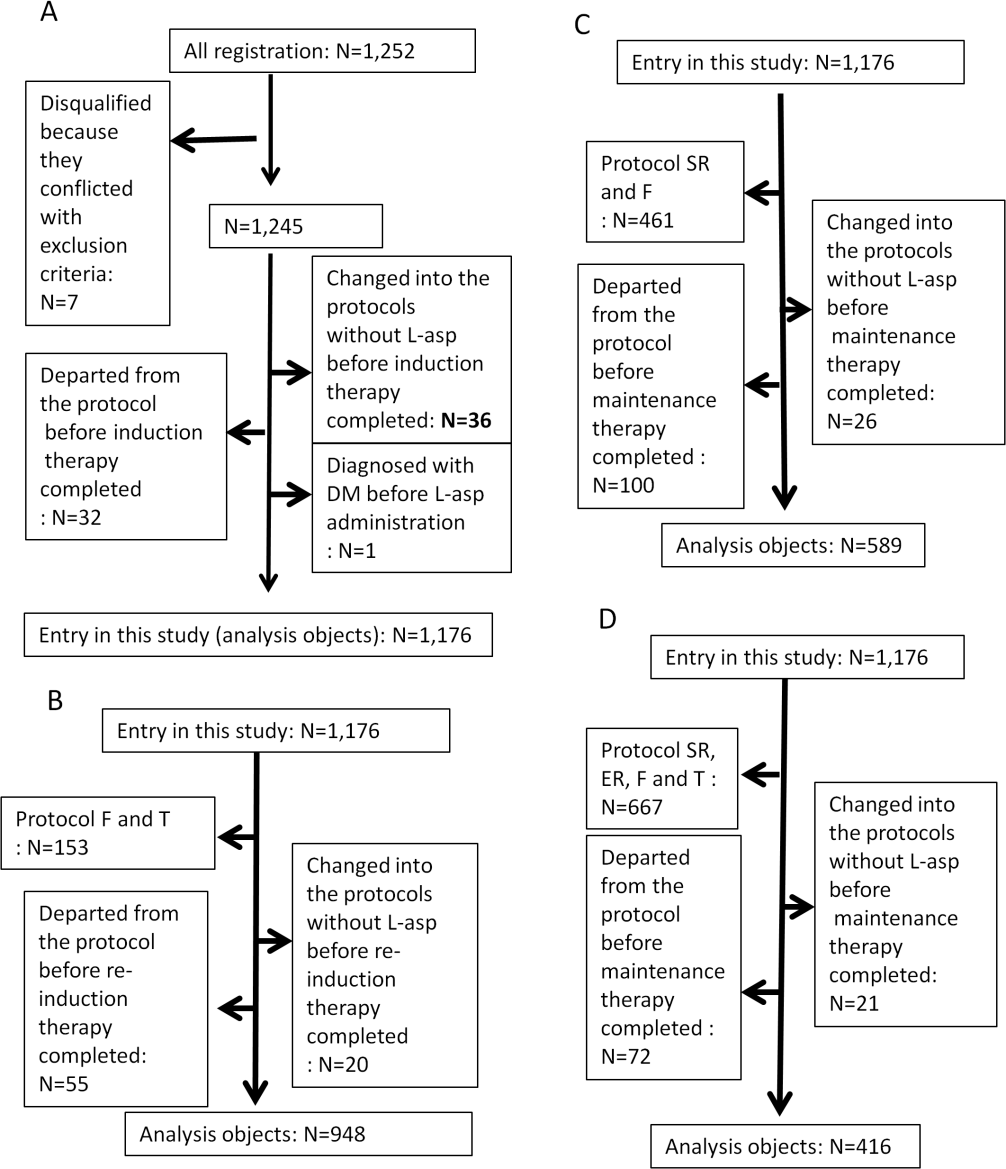
Design of the study and patient flow. (A) during the entire protocol and the induction phase. No height and weight data were recorded on the case report form at the Data Center for 72 cases. The remaining 1,104 cases were used for obesity analysis. (B) No height and weight data were recorded on the case report form at the Data Center for 51 cases during the re-induction phase. The remaining 897 cases were used for obesity analysis. (C) No height and weight data were recorded on the case report form at the Data Center for 37 HR, ER, and T patients during the maintenance phase. The remaining 552 cases were used for obesity analysis.

A total of 1,252 patients (aged 1–18 years) with newly diagnosed ALL were enrolled in the JACLS ALL-02 study from April 2002 to May 2008 [6, 18]. The median follow up period of our patients was 62 months. Patients with Ph+ ALL, mature B ALL, infant ALL, and natural killer cell leukaemia were excluded. Participants were stratified into standard risk (SR), high risk (HR), extremely high risk (ER), T cell type ALL (T), and induction failure (F) groups according to patient characteristics (**S1 Fig)**. Seventy-six patients were removed from the analysis due to the reasons described in **Fig 1A.** The characteristics of the remaining 1,176 patients (SR = 386, HR = 509, ER = 128, T = 78, and F = 75) were then analyzed (**Table 1)**.

**Table 1:** Characteristics of the 1,176 patients.

Features at diagnosis			Risk group					
			SR	HR	ER	T	F	Total
			n = 386	n = 509	n = 128	n = 78	n = 75	n = 1,176
Age (year)		Average ± SD (median)	4.1 ± 2.1 (4)	6.3 ± 4.3 (5)	7.0 ± 4.2 (6)	8.0 ± 3.5 (8)	7.7 ± 4.4 (7)	5.8 ± 3.9 (5)
at onset		< 10	386	370	89	53	47	945
		≥ 10	0	139	39	25	28	231
Sex		Female	195	232	60	11	28	526
		Male	191	277	68	67	47	650
Immuno-phenotype		B-cell	386	509	98	0	39	1,032
		Mixed	0	0	25	0	13	38
		T-cell	0	0	0	78	21	99
		Undifferentiated	0	0	5	0	2	7
Leukocyte count		Range (median)	0.37–9.98 (3.70)	0.43–401 (13.2)	1.00–420 (19.1)	0.72–819 (54.4)	1.10–816 (31.3)	0.72–819 (7.50)
at onset (× 10^9^/L)		< 20	386	317	69	26	31	829
		≥ 20-< 50	0	115	31	11	18	175
		≥ 50	0	77	28	41	26	172
Chromosome/		Normal karyotype	189	203	56	44	28	520
Genetic abnormality		Hyperdiploid	99	97	17	1	2	216
		*ETV6-RUNX1*	87	88	17	0	3	195
		*TCF3-PBX1*	0	68	8	0	2	78
Extramedullary disease		Present	0	18	5	9	5	37
Obesity	BMI	< 22%	361	461	114	68	63	1,067
		≥ 22%	3	19	8	2	5	37
	BMIp	< 85%	324	398	100	58	59	939
		≥ 85%	40	82	22	12	9	165
	Obesity index	< 20%	353	444	111	64	63	1,035
		≥ 20%	11	36	11	6	5	69
	No data		22	29	6	8	7	72
Down syndrome		Present	6	10	5	0	2	23

ER, extremely high risk; F, induction failure; HR, high risk; L, liter; SD, standard deviation; SR, standard risk

T, T-cell type; n, number; SD, standard deviation.

The L-asp administration schedule according to the JACLS ALL-02 protocol is summarized in **Tables A-E in S1 File**. The naïve *E*. *coli* L-asp preparation, i.e., Leunase (Kyowa Hakko Kirin, Tokyo, Japan) was used because PEG asparaginase has not been approved in Japan. Generally, L-asp was administered with prednisolone (PSL) (40 mg/m^2^), not dexamethasone (DEX), in this protocol. Protracted administration of L-asp in maintenance phase was employed in HR, ER and T-ALL protocol.

### Evaluation of risk factors for L-asp-related hyperglycemia

During the protocol study, adverse events were assessed according to National Cancer Institute (NCI)-Common Terminology Criteria for Adverse Events (CTCAE) version 2.0 and reported to the Data Center. L-asp-related hyperglycemia was defined as grade 3 or 4 hyperglycemia (serum glucose>250 mg/dl) occurring during L-asp treatment. Hyperglycemia due to L-asp-related pancreatitis was excluded. The risk factors for L-asp-related hyperglycemia were analyzed during the entire protocol: the induction phase, the re-induction phase, and the maintenance phase for HR, ER and T-ALL patients (**Fig 1A–1C**). The relationship between obesity and L-asp-related hyperglycemia was analyzed in 1,104 patients only due to a lack of height and weight data for 72 patients.

The characteristics of the 72 patients who lacked data did not differ from those of the remaining patients except for leukocyte count at onset and central nervous system status (**Table F in S1 File**).

Obesity was evaluated according to body mass index (BMI; cut off >22 kg/m^2^), BMI percentile (BMIp; cut off >85% (Ogden *et al*, 2010)), and obesity index (OI; cut off >20%). BMI was calculated as follows: body weight (kg)/{height (m)}^2^. The exclusive software (NordiFIT ver 3.0; Novo Nordisk, Denmark) was used to calculate BMIp. OI was calculated using the formulae provided by the Japanese Society for Paediatric Endocrinology (**Table G in S1 File**). The obesity rate according to the age group in this cohort is shown in **S2 Fig**.

### Statistical analysis

Multiple logistic regression model was used to investigate risk factors that were associated with L-asp-related hyperglycaemia, A factor was included in the model if the two-tailed P value for its univariate association with L-asp-related hyperglycaemia was P<0.05. Factors that were significant on multivariable analysis (P<0.05) were included in the final model; results were reported using adjusted odds ratios with 95% confidence intervals (95% CIs). Other comparisons were performed using the **χ** ^**2**^, Fisher’s exact, and Mann-Whitney U tests as appropriate. P <0.05 were considered significant.

## Results

### Clinical characteristics of patients who developed L-asp-related hyperglycemia

The clinical characteristics of the patients who developed L-asp-related hyperglycemia are summarized in **Table 2**. Sixty-nine of 1,176 (5.9%) patients experienced L-asp-related hyperglycemia and the hyperglycemic patients were significantly older (9.3 ± 4.1 vs 5.6 ± 3.8 years, P<0.01). Sex, initial WBC counts, immunophenotype and the presence of extramedullary disease were not statistically different between two groups. Down syndrome tends to be more in hyperglycemic patients, although it is not statistically significant (P = 0.05). A total of 75 L-asp related hyperglycemia events occurred in 69 patients. The grade, therapeutic phase, and risk group of the 75 events are summarized in **Table H in S1 File**. Fifty and 25 events were classified as grade 3 and grade 4 hyperglycemia, respectively. Seventeen (22.7%) events developed during the induction phase, 11 (14.7%) during the re-induction phase, 45 (60%) during the maintenance phase, and two during other phases. Forty-seven of 75 events (62.7%) occurred in the HR group treated with protracted administration of L-asp in maintenance phase. Three of the 69 patients (two HR and one T) switched to a protocol that did not contain L-asp because of severe hyperglycemia that developed during the maintenance phase.

**Table 2:** Comparison of patient characteristics with or without hyperglycemia (69 cases *vs*. 1,107 cases).

Feature at diagnosis		Category	Number of patients		
			With hyperglycemia	Without hyperglycemia	p-value
			n = 69	n = 1,107	
Age at onset (year)		Average ± SD (median)	9.3 ± 4.1 (10)	5.6 ± 3.8 (4)	< 0.01
Sex		Female	34	492	0.43
		Male	35	615	
Immunophenotype		B-cell	62	970	0.99
		Mixed	2	36	
		T-cell	5	94	
		Undifferentiated	0	7	
Leukocyte count at onset (× 10^9^/L)		Range (median)	0.60–509 (7.40)	0.37–819 (7.50)	0.41
Extramedullary disease		Absent	65	1,074	0.34
		Present	4	33	
Obesity	BMI	< 22%	59	1,008	< 0.01
		≥ 22%	8	29	
	BMIp	< 85%	50	889	0.01
		≥ 85%	17	148	
	Obesity index	< 20%	57	978	< 0.01
		≥ 20%	10	59	
	No data		2	72	
Down syndrome		Absent	65	1,088	0.05
		Present	4	19	

### Risk factors for L-asp-related hyperglycemia

Univariate analysis revealed that L-asp-related hyperglycemia correlated with older age (older than 5 years, 10 years and 15 years), type of protocol (HR and ER), higher BMI (≥22 kg/m^2^), higher BMIp (≥85%), higher OI (≥20%) and Down syndrome (P<0.05; **Table 3**), which are consistent with previous reports [15, 16, 19, 20]. Because more than half events developed in maintenance phase, univariate analysis was also performed for L-asp-related hyperglycemia during the induction, re-induction, and maintenance phases (**Table 3**). The analysis also showed that older age (older than 10 and 15 years) and higher BMI (≥22 kg/m^2^) correlated with L-asp-related hyperglycemia during the induction phase (P<0.05). Type of protocol was not associated with L-asp related hyperglycemia in induction phase just because the dose and schedule of L-asp administration was the same in all risk groups. Univariate analysis also showed that older age (older 5 and 10 years), female patient, higher BMI, and higher OI also correlated with hyperglycemia during the maintenance phase (P<0.05), but did not show any significant risk factors related to L-asp-related hyperglycemia in the re-induction phase, partly because the number of events (n = 11) was small. Multivariate analysis revealed that older age (older than10 years) was associated with L-asp-related hyperglycemia during the entire treatment and the maintenance phase. However, BMI (≥22 kg/m^2^) was not associated with L-asp-related hyperglycemia (**Table 4**). These findings suggested that protracted administration of L-asp with PSL was risk factor of hyperglycemia in the patients older than 10 years irrespective of obesity. On the other hand, either older age or higher BMI was not associated with L-asp-related hyperglycemia in induction phase, partly because the number of events was small (n = 17) (**Table 4**). However, 14 of 17 patients were older than 10 years and only two of 17 patients showed higher BMI.

**Table 3:** Univariate analysis of L-asp-related hyperglycemia.

		During entire treatment			Induction phase			Maintenance phase		
		Odds ratio	95% CI	p-value	Odds ratio	95% CI	p-value	Odds ratio	95% CI	p-value
Age at onset (yrs)										
	1–4 vs 5–18	1.000			1.000			1.000		
		4.534	2.441–8.422	<0.0001	1.330	1.330–3.500	0.564	11.157	3.323–37.462	<0.0001
	1–9 vs 10–18	1.000			1.000			1.000		
		5.442	3.308–8.953	<0.0001	3.731	1.424–9.778	0.0074	7.299	3.882–15.247	<0.0001
	1–14 vs 15–18	1.000			1.000			1.000		
		3.656	1.455–9.183	0.006	7.629	2.082–27.954	0.002	1.685	0.493–5.759	0.405
Sex										
	female	1.000			1.000			1.000		
	male	0.824	0.506–1.340	0.4342	1.158	0.432–3.453	0.7065	0.519	0.281–0.957	0.0358
Risk										
	SR	1.000			1.000			-	-	-
	HR	3.670	1.761–7.646	0.0005	0.628	0.217–2.630	0.6601	1.000		
	ER	4.735	1.974–11.361	0.0005	2.043	0.434–7.825	0.4069	1.001	0.449–2.234	0.9981
	F	1.148	0.243–5.421	0.8620	-	-	-	-	-	-
	T	2.264	0.679–7.547	0.1833	1.667	0.330–8.415	0.5363	0.286	0.067–1.216	0.0902
BMI										
	< 22%	1.000			1.000			1.000		
	≥ 22%	4.713	2.064–10.761	0.0002	6.636	1.822–24.177	0.0041	6.716	2.385–18.913	0.0003
BMI percentile										
	< 85%	1.000			1.000			1.000		
	≥ 85%	2.042	1.147–3.637	0.0153	2.414	0.839–6.945	0.1021	1.931	0.953–3.911	0.0677
Obesity index										
	< 20%	1.000			1.000			1.000		
	≥20%	2.908	1.413–5.984	0.0037	3.315	0.930–11.823	0.0647	2.805	1.160–6.783	0.0221
Down syndrome										
	Absent	1.000			1.000			1.000		
	Present	3.286	1.086–9.946	0.0352	2.975	0.378–23.430	0.3005	3.853	0.755–19.671	0.1049

BMI, body mass index; CI, Confidence interval; ER, extremely high risk; F, induction failure; HR, high risk; SD, standard deviation; SR, standard risk; T, T-cell type.

**Table 4:** Multivariate analysis of L-asp-related hyperglycemia.

		During entire treatment			Induction phase			Maintenance phase		
		Odds ratio	95% CI	p-value	Odds ratio	95% CI	p-value	Odds ratio	95% CI	p-value
Age at onset (yrs)										
	< 10	1.000			1.000			1.000		
	≥ 10	3.824	2.092–6.988	<0.0001	2.796	0.970–8.062	0.0570	6.415	3.116–13.205	<0.0001
Sex										
	Female							1.000		
	male							0.567	0.291–1.104	0.0949
Risk										
	SR	1.000			1.000			1.000		
	HR	1.801	0.760–4.267	0.1815	-	-	-	-	-	-
	ER	2.292	0.851–6.172	0.1008	-	-	-	-	-	-
	T	0.538	0.103–2.813	0.4624	-	-	-	-	-	-
	F	1.456	0.391–5.423	0.5775	-	-	-	-	-	-
BMI										
	< 22%	1.000			1.000			1.000		
	≥ 22%	1.813	0.727–4.522	0.2022	3.583	0.867–14.804	0.0779	2.597	0.842–8.015	0.0969

BMI, body mass index; CI, Confidence interval; ER, extremely high risk; F, induction failure; HR, high risk; SD, standard deviation; SR, standard risk; T, T-cell type.

### L-asp-related hyperglycemia does not affect prognosis

To evaluate the prognostic significance of L-asp-related hyperglycemia, we performed univariate and multivariate analyses of HR patients (n = 416). Univariate analysis revealed that older age (≥10) and development of L-asp-related hyperglycemia during the maintenance phase were associated with poorer event-free survival (EFS). However, multivariate analysis revealed that only older age (≥10) was associated with poor EFS (**Table 5**).

**Table 5:** Univariate and multivariate analyses of outcomes for patients in the high risk group.

		Univariate analysis				Multivariate analysis			
		Odds ratio	95%CI_low	95%CI_high	p-value	Odds ratio	95%CI_low	95%CI_high	p-value
Hyperglycaemia in induction phase									
	Absent	1.000				1.000			
	Present	5.953	0.525	67.446	0.1498	-	-	-	-
Hyperglycemia in re-induction phase									
	Absent	1.000				1.000			
	Present	< 0.001	< 0.001	> 999.999	0.9857	-	-	-	-
Hyperglycemia in maintenance phase									
	Absent	1.000				1.000			
	Present	3.413	1.362	8.557	0.0088	2.187	0.807	5.929	0.1242
Age at onset									
	< 10	1.000				1.000			
	≥ 10	3.060	1.478	6.332	0.0026	2.514	1.145	5.516	0.0215
Sex									
	Female	1.000				1.000			
	Male	1.630	0.769	3.456	0.2022	-	-	-	-
BMI									
	< 22%	1.000				1.000			
	≥ 22%	2.684	0.554	13.004	0.2200	-	-	-	-
BMI percentile									
	< 85%	1.000				1.000			
	≥ 85%	0.886	0.328	2.396	0.8122	-	-	-	-
Obesity index									
	< 20%	1.000				1.000			
	≥ 20%	0.921	0.208	4.086	0.9142	-	-	-	-
Down syndrome									
	Absent	1.000				1.000			
	Present	3.922	0.758	20.283	0.1031	-	-	-	-

BMI, body mass index; CI, Confidence interval

## Discussion

Previous studies of pediatric ALL patients show that older age (≥10 years) at disease onset and obesity are risk factors for L-asp-related hyperglycemia, especially during the induction phase [15, 16, 19, 20]. Pui et al retrospectively performed multivariate analysis for 421 children treated at St. Jude Children’s Hospital and identified four independent risk factors for the development of L-asp-related hyperglycemia during induction therapy: 1) age >10 years, 2) obesity (defined as ≥20% over ideal weight), 3) Down syndrome, and 4) having a first- or second-degree relative with diabetes mellitus [15]. Sonabend et al also used univariate analysis to identify four risk factors for the development of overt hyperglycemia (serum glucose>200 mg/dl) during the induction phase: 1) age >10 years, 2) BMIp ≥85%, 3) high risk according to the National Cancer Institute, and 4) use of prednisolone [20]. Lowas et al performed multivariate analysis on 33 (20.3%) of 162 patients who developed transient hyperglycemia (BS >200 mg/dl) during the induction phase and showed that older age (≥10 years) and higher BMIp (≥85%) were independent risk factors for developing transient hyperglycemia [19].

Here we analyzed 1,176 pediatric ALL patients treated according to the JACLS ALL-02 protocol. This is the largest cohort in which L-asp-related hyperglycemia has been investigated. We found that L-asp-related hyperglycemia occurred in 69 of 1,176 (5.9%) patients. Multivariate analysis identified older age (≥10 years) as the sole independent risk factor for L-asp-related hyperglycemia. The unique finding of this study was that obesity was not an independent risk factor (**Table 4**). In our protocol, none of the SR patients (who did not receive L-asp during the maintenance phase) were older than 10 years-of-age. Thus, the high incidence of L-asp-related hyperglycemia in older patients may be associated with prolonged use of L-asp combined with prednisolone during the maintenance phase (**Tables A-E in S1 File**), as 45 of 75 events (60%) occurred during this phase (**Table H in S1 File**). Accordingly, multivariate analysis identified a strong correlation between older age (≥10 years) and L-asp-related hyperglycemia during the maintenance phase (P<0.01, **Table 4**). Because protracted administration of L-asp in maintenance phase was not popular in other pediatric ALL protocols, hyperglycemia in maintenance phase seemed to be peculiar in our protocol. Thus, current national protocol for pediatric ALL does not employ protracted administration of L-asp in maintenance phase in Japan. On the other hand, the overall incidence of L-asp-related hyperglycemia during the induction phase was 1.4% (17 of 1,176), which was lower than those in the previous reports (10–15%) [15,16,19,20]. Relatively low dose of concomitant PSL (40 mg/m^2^) use and no administration of DEX might explain the low frequency of L-asp related hyperglycemia in induction phase in this study.

L-asp inhibits insulin synthesis by the pancreatic β cells, and L-asp-related hyperglycemia may occur readily in patients with insulin resistance [21]. Insulin resistance increases during adolescence partly because of increased growth hormone secretion [16, 22–24], which may explain why L-asp-related hyperglycemia occurs more often in older pediatric patients. Obesity is also related to insulin resistance through increased secretion of adipocytokines such as leptin and adiponectin [25, 26]. Increased visceral fat contributes to insulin resistance by decreasing blood adiponectin levels [26]. Because the pediatric standard of obesity according to BMI changes substantially with age and height, OI or BMIp may be more accurate indicators of obesity in a pediatric cohort [27]. Thus, we used these measures to analyze the effect of obesity more precisely in this study. However, multivariate analysis revealed that none of these measures of obesity were associated with L-asp-related hyperglycemia. The reason why obesity was not associated with occurrence of L-asp-related hyperglycemia is unclear. In this analysis, we evaluated three kinds of measures related to obesity; BMI, BMIp and OI, however, in all evaluations, there were clearly low frequency of obese patients in our cohort (**Tables 1 and 2**). Thus, the small number of the obese patients might cause different result from those in previous studies. Considering insulin resistance is deeply associated with L-asp-related hyperglycemia, the limitation of this study is that we could not evaluate serum biomarker of insulin resistance such as leptin, adiponectin, GH and IGF1. Because the age or BMI is just surrogate marker of insulin resistance, we should use more reliable biomarker(s) to evaluate personal insulin resistance in these studies.

Previous studies demonstrate that older age is a risk factor for various adverse events associated with L-asp treatment for pediatric ALL, including pancreatitis, thrombotic complications, hypertriglyceridemia, and hyperglycemia [28, 29]. However, pediatric protocols are recommended worldwide for AYA with ALL, as several recent studies show that pediatric protocols with intensive L-asp therapy are associated with EFS and disease-free survival in AYA patients with ALL [7, 8]. Although retrospective studies show that the prognosis for AYAs with ALL treated with a pediatric protocol is excellent [30], L-asp-related side effects should be monitored carefully in this age group.

Finally, we found that multivariate analysis revealed that L-asp-related hyperglycemia did not correlate with poor prognosis (**Table 5**). The excellent supportive care in our study ensured that most of the patients (66 of 69, 95.7%) completed the protocol. A univariate analysis performed by Sonabend et al showed that pediatric ALL patients with overt hyperglycemia (serum glucose > 200 mg/dl) had a 6.2 times greater risk of death [20]. Although they indicated that the relationship between hyperglycemia and death was unclear, this may be due to a high rate of NCI high risk patients among hyperglycemic group, suggesting that hyperglycemia might be just confounding factor for poor outcome.

In summary, we analyzed 1,176 pediatric ALL patients to identify risk factors for L-asp-related hyperglycemia. Multivariate analysis identified older age at onset (≥10 years) as the sole independent risk factor, suggesting that prolonged administration of L-asp combined with prednisolone may cause increased hyperglycemia in patients ≥10 years-of-age due to age-related insulin resistance. However, obesity (as measured by BMI, BMIp, and OI) was not correlated with L-asp-related hyperglycemia. These findings suggest that careful monitoring of hyperglycemia is essential when treating AYA with ALL, irrespective of obesity, using a pediatric protocol that includes intensive L-asp therapy.

## Supporting Information

S1 Fig Risk classification according to the JACLS ALL-02 protocol. ALL, acute lymphoblastic leukaemia; AUL, Acute undifferentiated leukaemia; CNS, central nervous system; ER, extremely high risk; F, induction failure; HR, high risk; IT, intrathecal therapy; M1, blasts < 5% in bone marrow; M2, 5% ≤ blasts < 25% in bone marrow; M3, blasts ≥ 25% in bone marrow; MTX, methotrexate; PGR, PSL good response; Ph-ALL, Philadelphia chromosome (t(9;22))-positive ALL; PPR, PSL poor response; PSL, prednisolone; SR, standard risk; T T cell type ALL; WBC, white blood cell.
(TIF)

S2 Fig Obesity rate according to the age in this study. Total number is 1,104, which has the data of height and weight. The rate of obesity is roughly around 10%, and at the most 20%. BMI, body mass index, BMIp, BMI percentile; n, number; OI, obesity index.
(TIF)

S1 File JACLS ALL-02 standard risk (SR) protocol (Table A), JACLS ALL-02 high risk (HR) protocol (CNS negative) (Table B), JACLS ALL-02 extremely high risk (ER) protocol (CNS negative) (Table C), JACLS ALL-02 T-cell protocol (CNS negative) (Table D), JACLS ALL-02 F protocol (CNS negative) (Table E), Comparison of patient characteristics with and without height and weight data (Table F), Formulae used to calculate the obesity index (Table G), Development and severity of hyperglycemia according to patient risk (Table H). (DOCX)
