# Supplementary figures and images for: Protracted Administration of L-Asparaginase in Maintenance Phase Is the Risk Factor for Hyperglycemia in Older Patients with Pediatric Acute Lymphoblastic Leukemia
Source: PLoS One. 2015 Aug 28;10(8):e0136428. doi: 10.1371/journal.pone.0136428 (PMC4552641; doi:10.1371/journal.pone.0136428)

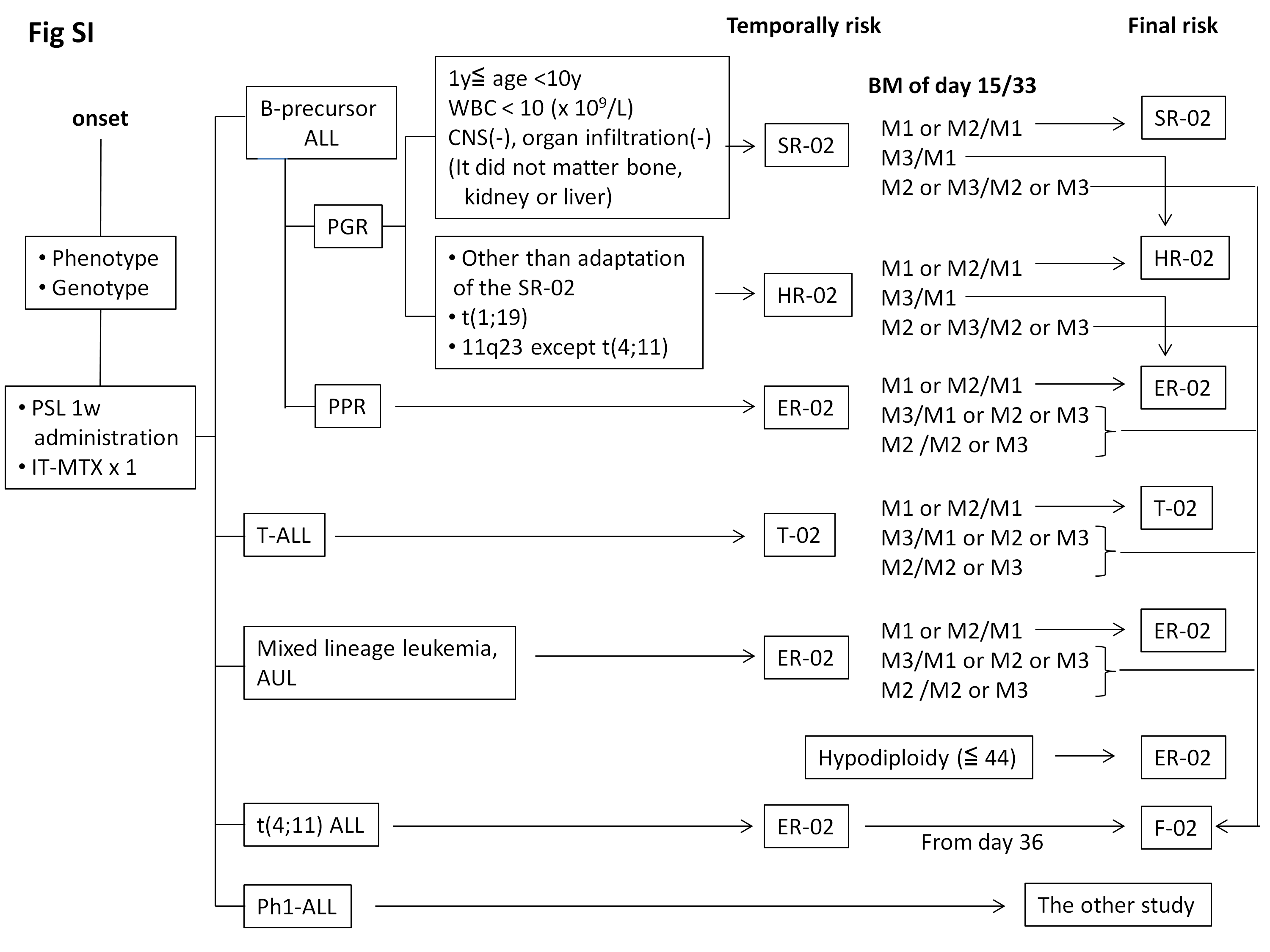

Supplement: S1 Fig — ALL, acute lymphoblastic leukaemia; AUL, Acute undifferentiated leukaemia; CNS, central nervous system; ER, extremely high risk; F, induction failure; HR, high risk; IT, intrathecal therapy; M1, blasts < 5% in bone marrow; M2, 5% ≤ blasts < 25% in bone marrow; M3, blasts ≥ 25% in bone marrow; MTX, methotrexate; PGR, PSL good response; Ph-ALL, Philadelphia chromosome (t(9;22))-positive ALL; PPR, PSL poor response; PSL, prednisolone; SR, standard risk; T T cell type ALL; WBC, white blood cell. (TIF) [file pone.0136428.s001.TIF]

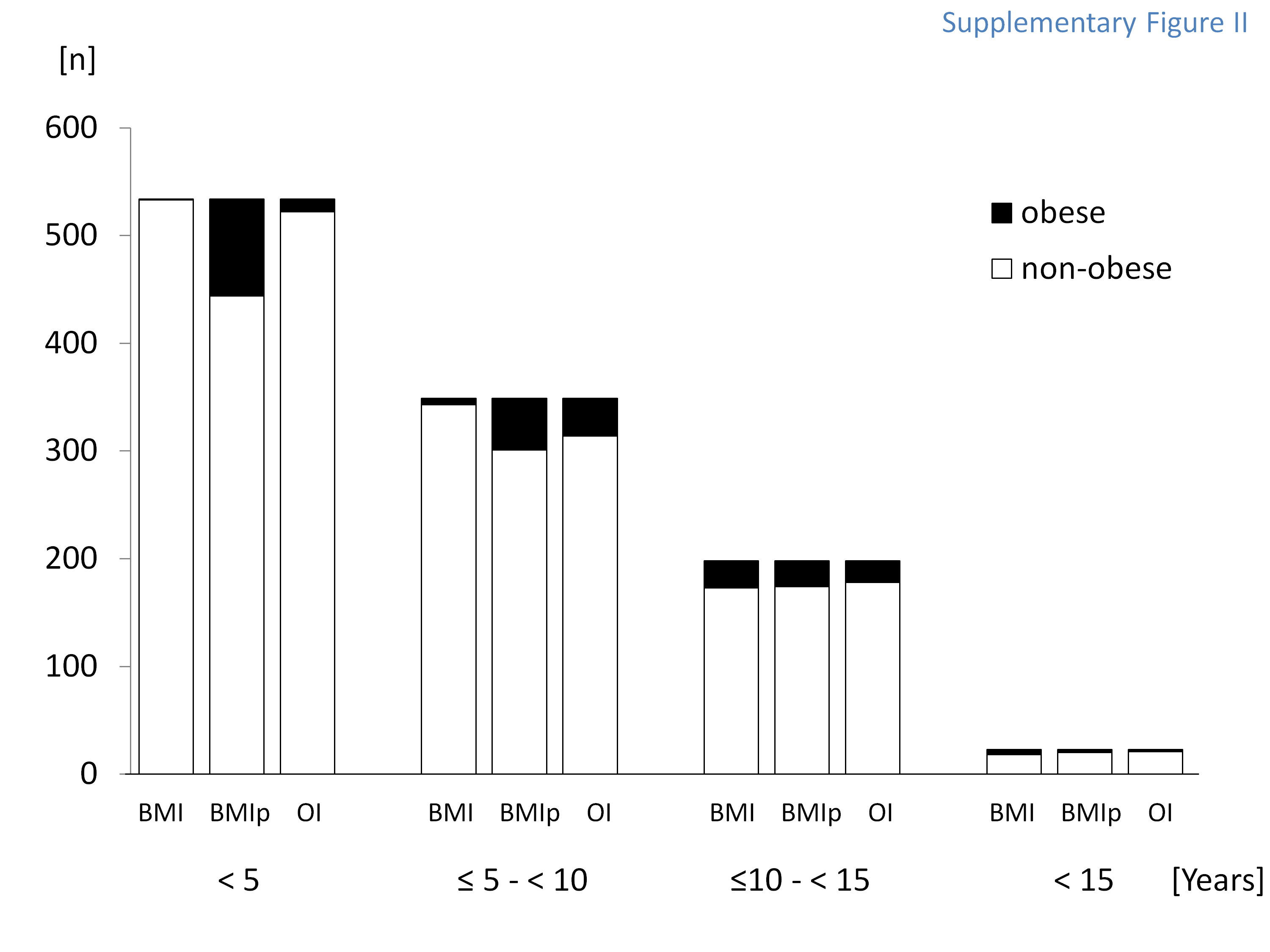

Supplement: S2 Fig — Total number is 1,104, which has the data of height and weight. The rate of obesity is roughly around 10%, and at the most 20%. BMI, body mass index, BMIp, BMI percentile; n, number; OI, obesity index. (TIF) [file pone.0136428.s002.TIF]
