# Supplementary material for: Protracted Administration of L-Asparaginase in Maintenance Phase Is the Risk Factor for Hyperglycemia in Older Patients with Pediatric Acute Lymphoblastic Leukemia
Source: PLoS One. 2015 Aug 28;10(8):e0136428. doi: 10.1371/journal.pone.0136428 (PMC4552641; doi:10.1371/journal.pone.0136428)
Supplement: S1 File — (DOCX) [file pone.0136428.s003.docx]

| **Table A.** JACLS ALL-02 standard risk (SR) protocol | | |
| --- | --- | --- |
| Drugs | Single or daily dose | Days given |
| Induction Therapy (Weeks 1-5) |  |  |
| Dexamethasone (1 hr div) | 10 mg/m^2^ | 8-14 (× 7 days) |
| **L-asparaginase (4 hr div or im)** | **6,000 U/m^2^** | **15, 17, 19, 22, 24, 26 (× 6 days)** |
| Pirarubicin (1 hr div) | 20 mg/m^2^ | 8 and 9 |
| Prednisolone (1 hr div) | 15-60 mg/m^2^ | 1-7 (× 7 days) |
| Prednisolone (po) | 40 mg/m^2^  10 mg/m^2^ | 15-28 (× 14 days)  29-31 (× 3days) |
| Vincristine (iv) | 1.5 mg/m^2^  (max 2.0 mg) | 8, 15, 22 and 29 (× 4 days) |
| Methotrexate (it) | ^a^ | 1 |
| triple intrathecal therapy ^b^ | ^c^ | 8 and 22 |
| Consolidation Therapy (Weeks 6-8): random allocation whether arm A or B | | |
| Arm A | |  |
| Cyclophosphamide (1 hr div) | 750 mg/m^2^ | 36 and 43 |
| Cytosine arabinoside (1 hr div) | 75 mg/m^2^ | 36-41, 43-48 (× 12 days) |
| 6-mercaptopurone (po) | 50 mg/ m^2^ | 36-49 (× 14 days) |
| triple intrathecal therapy ^b^ | ^c^ | 36 and 43 |
| Arm B |  |  |
| Cyclophosphamide (1 hr div) | 500 mg/m^2^ | 36, 38 and 40 (× 3 days) |
| Cytosine arabinoside (24 hr civ) | 100 mg/m^2^ | 36-40 (× 5 days) |
| Dexamethasone (1 hr div) | 10 mg/m^2^ | 36-40 (× 5 days) |
| triple intrathecal therapy ^b^ | ^c^ | 36 and 43 |
| Sanctuary Therapy (Weeks 9-10) |  |  |
| Methotrexate (24 hr civ) | 3000 mg/m^2^ | 57 and 64 |
| triple intrathecal therapy ^b^ | ^c^ | 58 and 65 |
| Re-induction therapy (Weeks 11-14) | |  |
| **L-asparaginase (im)** | **6,000 U/m^2^** | **71, 73, 75, 78, 80 and 82 (x 6 days)** |
| Pirarubicin (1 hr div) | 25 mg/m^2^ | 71 and 78 |
| Prednisolone (po) | 40 mg/m^2^  10 mg/m^2^ | 71-84 (× 14 days)  93-95 (× 3 days) |
| Vincristine (iv) | 1.5 mg/m^2^ (max 2.0 mg) | 71, 78 and 85 (× 3 days) |
| triple intrathecal therapy ^b^ | ^c^ | 78 |
| Maintenance therapy (Weeks 15-102) | |  |
| 6-mercaptopurine (po) | 50 mg/m^2^ | Every day in Weeks 15-102 |
| Methotrexate (po) | 25 mg/m^2^ | The first days of the every week  (× 88 days) |
| Prednisolone (po) | 40 mg/m^2^ | During Weeks 18, 22, 26, 30, 34, 38, 42, 46, 50, 54, 58, 62, 66, 70, 74, 78, 82, 86, 90, 94, 98 and 102 (7 days × 22) |
| Vincristine (iv) | 1.5 mg/m^2^ (max 2.0 mg) | The first days of Weeks 18, 22, 26, 30, 34, 38, 42, 46, 50, 54, 58, 62, 66, 70, 74, 78, 82, 86, 90, 94, 98 and 102  (× 22 days) |
| triple intrathecal therapy ^b^ | ^c^ | The first days of Weeks 15, 23, 31, 39 and 47 (× 5 days) |

Abbreviations: civ, continuous intravenous infusion; div, intravenous infusion by drip; SR, standard risk; im, intramuscular infusion; it, intrathecal therapy; iv, intravenous infusion; JACLS, Japan Association of Childhood Leukemia Study; po, per oral.

^a^ 8 mg (1 year old), 10 mg (2 years old), 12 mg (over 3 years old); ^b^ methotrexate (MTX) + cytosine arabinoside (CA) + hydrocortisone (HDC); ^c^ MTX/CA/HDC: 8/20/15 mg (1 year old), 10/25/20 mg (2 years old), 12/30/25 mg (over 3 years old).

| **Table B**  JACLS ALL-02 high risk (HR) protocol (CNS negative) | | |
| --- | --- | --- |
| Drugs | Single or daily dose | Days given |
| Induction Therapy (Weeks 1-5) | |  |
| Cyclophospamide (1 hr div) | 1,200 mg/m^2^ | 10 |
| Dexamethasone (1 hr div) | 10 mg/m^2^ | 8-14 (× 7 days) |
| **L-asparaginase (4 hr div or im)** | **6,000 U/m^2^** | **15, 17, 19, 22, 24 and 26 (× 6 days)** |
| Pirarubicin (1 hr div) | 20 mg/m^2^ | 8 and 9 |
| Prednisolone (1 hr div) | 15-60 mg/m^2^ | 1-7 (× 7 days) |
| Prednisolone (po) | 40 mg/m^2^  10 mg/m^2^ | 15-28 (× 14 days)  29-31 (× 3 days) |
| Vincristine (iv) | 1.5 mg/m^2^  (max 2.0 mg) | 8, 15, 22 and 29 (× 4 days) |
| Methotrexate (it) | ^a^ | 1 |
| triple intrathecal therapy ^b^ | ^c^ | 8 and 22 |
| Consolidation Therapy (Weeks 6-9): Random allocation whether arm A or B | | |
| Arm A |  |  |
| Cyclophosphamide (1 hr div) | 750 mg/m^2^ | 36 and 43 |
| Cytosine arabinoside (1 hr div) | 75 mg/m^2^ | 36-41, 43-48 (× 12 days) |
| 6-mercaptopurone (po) | 50 mg/ m^2^ | 36-49 (× 14 days) |
| Pirarubicin (1 hr div) | 25 mg/m^2^ | 36 and 37 |
| triple intrathecal therapy ^b^ | ^c^ | 36 and 43 |
| Arm B |  |  |
| Cyclophosphamide (1 hr div) | 500 mg/m^2^ | 36, 38 and 40 (× 3 days) |
| Cytosine arabinoside (24 hr civ) | 100 mg/m^2^ | 36-40 (× 5 days) |
| Dexamethasone (1 hr div) | 10 mg/m^2^ | 36-40 (× 5 days) |
| Pirarubicin (1 hr div) | 25 mg/m^2^ | 36 and 37 |
| triple intrathecal therapy ^b^ | ^c^ | 36 and 43 |
| Sanctuary Therapy (Weeks 10-11) | |  |
| Methotrexate (24 hr civ) | 3000 mg/m^2^ | 64 and 71 |
| triple intrathecal therapy ^b^ | ^c^ | 65 and 72 |
| Re-induction therapy (Weeks 12-15) | |  |
| Cyclophosphamide (1 hr div) | 500 mg/m^2^ | 78 and 85 |
| **L-asparaginase (im)** | **6,000 U/m^2^** | **78, 80, 82, 85, 87 and 89 (× 6 days)** |
| Pirarubicin (1 hr div) | 25 mg/m^2^ | 78 and 85 |
| Prednisolone (po) | 40 mg/m^2^  10 mg/m^2^ | 78-91 (× 14 days)  92-94 (× 3 days) |
| Vincristine (iv) | 1.5 mg/m^2^ (max 2.0 mg) | 78, 85 and 92 (× 3 days) |
| triple intrathecal therapy ^b^ | ^c^ | 78 |
| Re-Consolidation Therapy (Weeks 16-19): The same arm as Consolidation Therapy | | |
| Arm A |  |  |
| Cyclophosphamide (1 hr div) | 750 mg/m^2^ | 106 and 113 |
| Cytosine arabinoside (1 hr div) | 75 mg/m^2^ | 106-111, 113-118 (× 12 days) |
| 6-mercaptopurone (po) | 50 mg/ m^2^ | 106-119 (× 14 days) |
| Pirarubicin (1 hr div) | 25 mg/m^2^ | 106 and 107 |
| triple intrathecal therapy ^b^ | ^c^ | 106 and 113 |
| Arm B |  |  |
| Cyclophosphamide (1 hr div) | 500 mg/m^2^ | 106, 108 and 110 (× 3 days) |
| Cytosine arabinoside (24 hr civ) | 100 mg/m^2^ | 106-110 (× 5 days) |
| Dexamethasone (1 hr div) | 10 mg/m^2^ | 106-110 (× 5 days) |
| Pirarubicin (1 hr div) | 25 mg/m^2^ | 106 and 107 |
| triple intrathecal therapy ^b^ | ^c^ | 106 and 113 |
| Maintenance therapy (Weeks 20-98) | |  |
| Cyclophosphamide (1 hr div) | 600 mg/m^2^ | The first days of Weeks 27, 47, 67 and 87 (× 4 days) |
| **L-asparaginase (im)** | **10,000 U/m^2^** | **The first days of Weeks 26, 27, 28, 36, 37, 38, 46, 47, 48, 56, 57, 58, 66, 67, 68, 76, 77, 78, 86, 87, 88, 96, 97 and 98 (× 24 days)** |
| Methotrexate (iv) | 150 mg/m^2^ | The first days of Weeks 20, 22, 24, 30, 32, 34, 40, 42, 44, 50, 52, 54, 60, 62, 64, 70, 72, 74, 80, 82, 84, 90, 92 and 94 (× 24 days) |
| 6-mercaptopurine (po) | 50 mg/m^2^ | During Weeks 20-23, 30-33, 40-43, 50-53, 60-63, 70-73, 80-83 and 90-93 (28 days × 8) |
| Pirarubicin (1 hr div) | 25 mg/m^2^ | The first days of Weeks 37, 57, 77 and 97 (× 4 days) |
| Prednisolone (po) | 40 mg/m^2^ | During Weeks 26-27, 36-37, 46-47, 56-57, 66-67, 76-77, 86-87 and 96-97 (14 days × 8) |
| Vincristine (iv) | 1.5 mg/m^2^ (max 2.0 mg) | On days same as L-asparaginase |
| triple intrathecal therapy ^b^ | ^c^ | The first days of Weeks 24, 34, 44, 54 and 64 (× 5 days) |

Abbreviations: civ, continuous intravenous infusion; CNS, central nerves system; div, intravenous infusion by drip; HR, high risk; im, intramuscular infusion; it, intrathecal therapy iv, intravenous infusion; JACLS, Japan Association of Childhood Leukemia Study; po, per oral.

^a^ 8 mg (1 year old), 10 mg (2 years old), 12 mg (over 3 years old); ^b^ methotrexate (MTX) + cytosine arabinoside (CA) + hydrocortisone (HDC); ^c^ MTX/CA/HDC: 8/20/15 mg (1 year old), 10/25/20 mg (2 years old), 12/30/25 mg (over 3 years old).

| **Table C**  JACLS ALL-02 extremely high risk (ER) protocol (CNS negative) | | |
| --- | --- | --- |
| Drugs | Single or daily dose | Days given |
| Induction Therapy (Weeks 1-5) | |  |
| Cyclophospamide (1 hr div) | 1,200 mg/m^2^ | 10 |
| Dexamethasone (1 hr div) | 10 mg/m^2^ | 8-14 (× 7 days) |
| **L-asparaginase (4 hr div or im)** | **6,000 U/m^2^** | **15, 17, 19, 22, 24 and 26 (× 6 days)** |
| Pirarubicin (1 hr div) | 20 mg/m^2^ | 8, 9 and 10 (× 3 days) |
| Prednisolone (1 hr div) | 15-60 mg/m^2^ | 1-7 (× 7 days) |
| Prednisolone (po) | 40 mg/m^2^  10 mg/m^2^ | 15-28 (× 14 days)  29-31 (× 3 days) |
| Vincristine (iv) | 1.5 mg/m^2^  (max 2.0 mg) | 8, 15, 22 and 29 (× 4 days) |
| Methotrexate (it) | ^a^ | 1 |
| triple intrathecal therapy ^b^ | ^c^ | 8 and 22 |
| Early Intensification (Weeks 6-9) | | |
| Cyclophosphamide (1 hr div) | 1,200 mg/m^2^ | 38 |
| Cytosine arabinoside (4 hr div) | 300 mg/m^2^ | 36-40 (× 5 days) |
| Etoposide (2 hr div) | 100 mg/ m^2^ | 36-40 (× 5 days) |
| Pirarubicin (1 hr div) | 25 mg/m^2^ | 37 and 39 |
| triple intrathecal therapy ^b^ | ^c^ | 36 and 43 |
| Consolidation Therapy A1 (Weeks 10-13) | |  |
| Cytosine arabinoside (3 hr div) | 2 g/m^2^ | 67-69 (four times in 3 days) |
| **L-asparaginase (4 hr div or im)** | **20,000 U/m^2^** | **69** |
| Methotrexate (12 hr div) | 3.0 g/m^2^ | 64 |
| Pirarubicin (1 hr div) | 25 mg/m^2^ | 67 |
| Prednisolone (po) | 40 mg/m^2^ | 64-69 (× 6 days) |
| Vincristine (iv) | 1.5 mg/m^2^ (max 2.0 mg) | 64 and 71 |
| triple intrathecal therapy ^b^ | ^c^ | 65 |
| Consolidation Therapy B1 (Weeks 14-17) | |  |
| Cyclophosphamide (1 hr div) | 1,200 mg/m^2^ | 94 |
| Cytosine arabinoside (4 hr div) | 300 mg/m^2^ | 92-96 (× 5 days) |
| Dexamethasone (1 hr div) | 100 mg/m^2^ | 92, 94 and 96 (× 3 days) |
| Etoposide (2 hr div) | 100 mg/ m^2^ | 92-96 (× 5 days) |
| Pirarubicin (1 hr div) | 25 mg/m^2^ | 93 and 95 |
| triple intrathecal therapy ^b^ | ^c^ | 92 |
| Consolidation Therapy A2 (Weeks 18-21) | |  |
| Cytosine arabinoside (3 hr div) | 2 g/m^2^ | 123-125 (four times in 3 days) |
| **L-asparaginase (4 hr div or im)** | **20,000 U/m^2^** | **125** |
| Methotrexate (12 hr div) | 3.0 g/m^2^ | 120 |
| Pirarubicin (1 hr div) | 25 mg/m^2^ | 123 |
| Prednisolone (po) | 40 mg/m^2^ | 120-125 (× 6 days) |
| Vincristine (iv) | 1.5 mg/m^2^ (max 2.0 mg) | 120 and 127 |
| triple intrathecal therapy ^b^ | ^c^ | 120 |
| Consolidation Therapy B2 (Weeks 22-25) | |  |
| Cyclophosphamide (1 hr div) | 1,200 mg/m^2^ | 150 |
| Cytosine arabinoside (4 hr div) | 300 mg/m^2^ | 148-152 (× 5 days) |
| Dexamethasone (1 hr div) | 100 mg/m^2^ | 148, 150 and 152 (× 3 days) |
| Etoposide (2 hr div) | 100 mg/ m^2^ | 148-152 (× 5 days) |
| Pirarubicin (1 hr div) | 25 mg/m^2^ | 149 and 151 |
| triple intrathecal therapy ^b^ | ^c^ | 148 |
| Re-induction therapy (Weeks 26-29) | |  |
| Cyclophosphamide (1 hr div) | 500 mg/m^2^ | 176 and 183 |
| **L-asparaginase (im)** | **6,000 U/m^2^** | **176, 178, 180, 183, 185 and 187  (× 6 days)** |
| Pirarubicin (1 hr div) | 25 mg/m^2^ | 176 and 183 |
| Prednisolone (po) | 40 mg/m^2^  10 mg/m^2^ | 176-189 (× 14 days)  190-192 (× 3 days) |
| Vincristine (iv) | 1.5 mg/m^2^ (max 2.0 mg) | 176, 183 and 190 (× 3 days) |
| triple intrathecal therapy ^b^ | ^c^ | 176 and 183 |
| Maintenance therapy (Weeks 30-108) | |  |
| Cyclophosphamide (1 hr div) | 600 mg/m^2^ | The first days of Weeks 37, 57, 77 and 97 (× 4 days) |
| **L-asparaginase (im)** | **10,000 U/m^2^** | **The first days of Weeks 36, 37, 38, 46, 47, 48, 56, 57, 58, 66, 67, 68, 76, 77, 78, 86, 87, 88, 96, 97, 98, 106, 107 and 108 (× 24 days)** |
| 6-mercaptopurine (po) | 50 mg/m^2^ | During Weeks 30-33, 40-43, 50-53, 60-63, 70-73, 80-83, 90-93 and 100-103 (28 days × 8) |
| Methotrexate (iv) | 150 mg/m^2^ | The first days of Weeks 30, 32, 34, 40, 42, 44, 50, 52, 54, 60, 62, 64, 70, 72, 74, 80, 82, 84, 90, 92, 94, 100, 102 and 104 (× 24 days) |
| Pirarubicin (1 hr div) | 25 mg/m^2^ | The first days of Weeks 47, 67, 87 and 107 (× 4 days) |
| Prednisolone (po) | 40 mg/m^2^ | During Weeks 36-37, 46-47, 56-57, 66-67, 76-77, 86-87, 96-97 and 106-107 (14 days × 8) |
| Vincristine (iv) | 1.5 mg/m^2^ (max 2.0 mg) | On days same as L-asparaginase |
| triple intrathecal therapy ^b^ | ^c^ | The first days of Weeks 34, 44, 54 and 64 (× 4 days) |

Abbreviations: civ, continuous intravenous infusion; CNS, central nerves system; div, intravenous infusion by drip; ER, extremely high risk; im, intramuscular infusion; it, intrathecal therapy iv, intravenous infusion; JACLS, Japan Association of Childhood Leukemia Study; po, per oral.

^a^ 8 mg (1 year old), 10 mg (2 years old), 12 mg (over 3 years old); ^b^ methotrexate (MTX) + cytosine arabinoside (CA) + hydrocortisone (HDC); ^c^ MTX/CA/HDC: 8/20/15 mg (1 year old), 10/25/20 mg (2 years old), 12/30/25 mg (over 3 years old).

| **Table D**  JACLS ALL-02 T-cell protocol (CNS negative) | | |
| --- | --- | --- |
| Drugs | Single or daily dose | Days given |
| Induction Therapy (Weeks 1-5) |  |  |
| Cyclophospamide (1 hr div) | 1,200 mg/m^2^ | 10 |
| Dexamethasone (1 hr div) | 10 mg/m^2^ | 8-14 (× 7 days) |
| **L-asparaginase (4 hr div or im)** | **6,000 U/m^2^** | **15, 17, 19, 22, 24, 26 (× 6 days)** |
| Pirarubicin (1 hr div) | 20 mg/m^2^ | 8 and 9 |
| Prednisolone (1 hr div) | 15-60 mg/m^2^ | 1-7 (× 7 days) |
| Prednisolone (po) | 40 mg/m^2^  10 mg/m^2^ | 15-28 (×14 days)  29-31 (× 3 days) |
| Vincristine (iv) | 1.5 mg/m^2^  (max 2.0 mg) | 8, 15, 22 and 29 (× 4 days) |
| Methotrexate (it) | ^a^ | 1 |
| triple intrathecal therapy ^b^ | ^c^ | 8 and 22 |
| Consolidation Therapy (Weeks 6-9) / Re-Consolidation Therapy (Weeks 15-18) | | |
| Cyclophosphamide (1 hr div) | 500 mg/m^2^ | 36, 38 and 40 / 99, 101 and 103  (×3 days, respectively) |
| Cytosine arabinoside (24 hr civ) | 100 mg/m^2^ | 36-42/99-105 (× 7 days, respectively) |
| Dexamethasone (1 hr div) | 10 mg/m^2^ | 36-42/99-105 (× 7 days, respectively) |
| Pirarubicin (1 hr div) | 25 mg/m^2^ | 36 and 37/99 and 100 |
| triple intrathecal therapy ^b^ | ^c^ | 36 and 43/99 and 106 |
| Sanctuary Therapy (Weeks 10-14) | |  |
| Cytosine arabinoside (24 hr civ) | 100 mg/m^2^ | 72-76 (× 5 days) |
| **L-asparaginase (4 hr div or im)** | **6,000 U/m^2^** | **73-77 (× 5 days)** |
| Methotrexate (24 hr civ) | 3000 mg/m^2^ | 64 and 71 |
| Prednisolone (po) | 40 mg/m^2^ | 71-77 (× 7 days) |
| triple intrathecal therapy ^b^ | ^c^ | 64 and 71 |
| Early Maintenance therapy (Weeks 19-36): WBC at onset<100 (× 10^9^/L) | | |
| **L-asparaginase (im)** | **10,000 U/m^2^** | **The first day of Weeks 19, 20, 22, 23, 25, 26, 28, 29, 31, 32, 34 and 35 (× 12 days)** |
| 6-mercaptopurine (po) | 50 mg/m^2^ | During Weeks 19-20, 22-23, 25-26, 28-29, 31-32 and 34-35  (14 days × 6) |
| Pirarubicin (1 hr div) | 25 mg/m^2^ | The first days of Weeks 19, 22, 28 and 31 (× 4 days) |
| Prednisolone (po) | 60 mg/m^2^ | During weeks 19, 22, 25, 28,31 and 34 (7 days × 6) |
| Vincristine (iv) | 1.5 mg/m^2^ (max 2.0 mg) | The first days of Weeks 19, 22, 25, 28, 31 and 34 (× 6 days) |
| triple intrathecal therapy ^b^ | ^c^ | The first days of Weeks 19 and 28 (× 5 days) |
| Maintenance therapy IA (Weeks 37-41, 47-51) | |  |
| 6-mercaptopurine (po) | 50 mg/m^2^ | During Weeks 37-40 and 47-50 (× 28 days, respectively) |
| Methotrexate (iv) | 150 mg/m^2^ | The first days of Weeks 37, 39, and 41 / 47, 49, and 51  (× 3 days, respectively) |
| triple intrathecal therapy ^b^ | ^c^ | 41 / 51 |
| Maintenance therapy IB (Weeks 42-46, 52-56) | |  |
| Cyclophospamide (1 hr div) | 600 mg/m^2^ | The first day of weeks 44/54 |
| **L-asparaginase (im)** | **10,000 U/m^2^** | **The first day of Weeks 43, 44 and 45/53, 54 and 55  (× 3 days, respectively)** |
| Prednisolone (po) | 40 mg/m^2^ | During the weeks 43-44 and the first day of week 45/During Weeks 53-54 and the first day of week 55  (× 15 days, respectively) |
| Vincristine (iv) | 1.5 mg/m^2^  (max 2.0 mg) | On days same as L-asparaginase |
| Maintenance therapy II (Weeks 57-104) | |  |
| 6-mercaptopurine (po) | 50 mg/m^2^ | Every day in Weeks 57-104 |
| Methotrexate (po) | 25 mg/m^2^ | The first days of the every week  (× 48 days) |

Abbreviations: civ, continuous intravenous infusion; div, intravenous infusion by drip; im, intramuscular infusion; it, intrathecal therapy; iv, intravenous infusion; JACLS, Japan Association of Childhood Leukemia Study; po, per oral; T, T cell type acute lymphoblastic leukaemia.

^a^ 8 mg (1 year old), 10 mg (2 years old), 12 mg (over 3 years old); ^b^ methotrexate (MTX) + cytosine arabinoside (CA) + hydrocortisone (HDC); ^c^ MTX/CA/HDC: 8/20/15 mg (1 year old), 10/25/20 mg (2 years old), 12/30/25 mg (over 3 years old).

| **Table E**  JACLS ALL-02 F protocol (CNS negative) | | |
| --- | --- | --- |
| Drugs | Single or daily dose | Days given |
| Re-Induction Therapy (Weeks 6-10) * This protocol starts from the sixth week based on the result of 1) bone marrow aspiration on Days 15 and 33 of the other protocols and 2) the chromosome. | | |
| Cytosine arabinoside (24 hr civ) | 500 mg/m^2^ | 36-38, 43-45 (× 6 days) |
| Etoposide (4 hr div) | 200 mg/ m^2^ | 43-45 (× 3 days) |
| Mitoxantrone (1 hr div) | 8 mg/m^2^ | 36-38 (× 3 days) |
| Prednisolone (po) | 40 mg/m^2^ | 36-38, 43-45 (× 6 days) |
| triple intrathecal therapy ^b^ | ^c^ | 8 and 22 |
| Consolidation Therapy A1 (Weeks 11-14)/Consolidation Therapy A1 (Weeks 19-22) | | |
| Cytosine arabinoside (24 hr civ) | 100 mg/m^2^ | 71-75 / 127-131 (× 5 days, respectively) |
| Dexamethasone (1 hr div) | 100 mg/m^2^ | 71, 73 and 75/127, 129 and 131 (× 3 days, respectively) |
| Etoposide (2 hr div) | 100 mg/ m^2^ | 71-75/ 127-131 (× 5 days, respectively) |
| Mitoxantrone (1 hr div) | 4 mg/m^2^ | 71-73/127-129 (× 3 days, respectively) |
| triple intrathecal therapy ^b^ | ^c^ | 71/127 |
| Consolidation Therapy B1 (Weeks 15-18)/Consolidation Therapy A1 (Weeks 23-26) | | |
| Cytosine arabinoside (24 hr civ) | 100 mg/m^2^ | 100-104/156-160  (× 5 days, respectively) |
| **L-asparaginase (4 hr div or im)** | **6,000 U/m^2^** | **101-105/157-161 (×5 days**, **respectively)** |
| Methotrexate (24 hr civ) | 3000 mg/m^2^ | 99/155 |
| Pirarubicin (1 hr div) | 25 mg/m^2^ | 100/156 |
| Prednisolone (po) | 40 mg/m^2^ | 99-105/155-161 (× 7 days, respectively) |
| Vincristine (iv) | 1.5 mg/m^2^ (max 2.0 mg) | 99/155 |
| triple intrathecal therapy ^b^ | ^c^ | 99/155 |
| Maintenance therapy (Weeks 27-105) | | |
| Cyclophosphamide (1 hr div) | 600 mg/m^2^ | The first days of Weeks 44, 64, 84 and 104 (× 4 days) |
| Etoposide (2 hr div) | 100 mg/ m^2^ | The first days of Weeks 34, 54, 74 and 94 (× 4 days) |
| Methotrexate (iv) | 150 mg/m^2^ | The first days Weeks 27, 29, 31, 37, 39, 41, 47, 49, 51, 57, 59, 61, 67, 69, 71, 77, 79, 81, 87, 89, 91, 97, 99 and 101 (× 24 days) |
| 6-mercaptopurine (po) | 50 mg/m^2^ | During Weeks 27-30, 37-40, 47-50, 57-60, 67-70, 77-80, 87-90 and 97-100 (28 days × 8) |
| Pirarubicin (1 hr div) | 25 mg/m^2^ | The first days of weeks 34, 44, 54, 64, 74, 84, 94 and 104 (× 8 days) |
| Prednisolone (po) | 40 mg/m^2^ | During weeks 33-34, 43-44, 53-54, 63-64, 73-74, 83-84, 93-94 and 103-104 (14 days × 8) |
| Vindesine (iv) | 3 mg/m^2^ (max 4.0 mg) | The first days of weeks 33, 34, 35, 43, 44, 45, 53, 54, 55, 63, 64, 65, 73, 74, 75, 83, 84, 85, 93, 94, 95, 103, 104 and 105 (× 48 days) |
| triple intrathecal therapy ^b^ | ^c^ | The first days of weeks 31, 41, 51 and 61 (× 4 days) |

Abbreviations: civ, continuous intravenous infusion; div, intravenous infusion by drip; F, induction failure; im, intramuscular infusion; it, intrathecal therapy; iv, intravenous infusion; JACLS, Japan Association of Childhood Leukemia Study; po, per oral; T, T cell type acute lymphoblastic leukaemia.

^a^ 8 mg (1 year old), 10 mg (2 years old), 12 mg (over 3 years old); ^b^ methotrexate (MTX) + cytosine arabinoside (CA) + hydrocortisone (HDC); ^c^ MTX/CA/HDC: 8/20/15 mg (1 year old), 10/25/20 mg (2 years old), 12/30/25 mg (over 3 years old).

| **Table F** Comparison of patient characteristics with and without height and weight data | | | | |
| --- | --- | --- | --- | --- |
| Feature at diagnosis | Category | Number of patients | |  |
|  |  | With data | Without data | p-value |
|  |  | n = 1,104 | n = 72 |  |
| Age at onset (year) | Average ± SD (median) | 5.9 ± 3.9 (5) | 5.4 ± 3.8 (4) | 0.28 |
|  | < 10 | 883 | 62 | 0.20 |
|  | ≥ 10 | 221 | 10 |  |
| Sex | Female | 488 | 38 | 0.16 |
|  | Male | 616 | 34 |  |
| Immunophenotype | B-cell | 972 | 60 | 0.27 |
|  | Mixed | 37 | 1 |  |
|  | T-cell | 88 | 11 |  |
|  | Undifferentiated | 7 | 0 |  |
| Down syndrome | Absent | 1,083 | 70 | 0.94 |
|  | Present | 21 | 2 |  |
|  |  | (n = 1,104) | (n = 72) |  |
|  |  |  |  |  |
| Hyperglycaemia during entire treatment | Absent | 1,037 | 70 | 0.37 |
|  | Present | 67 | 2 |  |
|  |  | (n = 1,104) | (n = 72) |  |
| Hyperglycaemia during induction phase | Absent | 1,087 | 72 | 0.58 |
|  | Present | 17 | 0 |  |
|  |  | (n = 897) | (n = 51) |  |
| Hyperglycaemia during re-induction phase | Absent | 886 | 51 | 0.90 |
|  | Present | 11 | 0 |  |
|  |  | (n = 552) | (n = 37) |  |
| Hyperglycaemia during maintenance phase of HR, ER and T-ALL | Absent | 509 | 35 | 0.97 |
|  | Present | 43 | 2 |  |
|  |  | (n = 390) | (n = 26) |  |
| Hyperglycaemia during Maintenance phase of HR | Absent | 357 | 24 | 0.83 |
|  | Present | 33 | 2 |  |
|  |  | (n = 1,104) | (n = 72) |  |
| Event related to poor prognosis | Absent | 919 | 62 | 0.53 |
|  | present | 185 | 10 |  |
|  |  |  |  |  |
|  |  |  |  |  |

Abbreviations: ALL, acute lymphoblastic leukaemia; ER, extremely high risk; HR, high risk; n, number; SD, standard deviation; T, T cell type ALL.

| **Table G** Formulae used to calculate the obesity index | | | |
| --- | --- | --- | --- |
| Male | < 6 years old | 70cm ≤ HT < 120cm | std BW = 0.00206X^2^ - 0.1166X + 6.5273 |
|  | ≥ 6 years old | 101cm ≤ HT < 140cm | std BW = 0.0000303882X^3^ - 0.00571495X^2^ + 0.508124X - 9.17791 |
|  |  | 140cm ≤ HT < 149cm | std BW = -0.000085013X^3^ + 0.0370692X^2^ - 4.6558X + 191.847 |
|  |  | 149cm ≤ HT < 184cm | std BW = -0.000310205X^3^ + 0.151159X^2^ - 23.6303X + 1231.04 |
| Female | < 6 years old | 70cm ≤ HT < 120cm | std BW = 0.00249X^2^ - 0.1858X + 9.0360 |
|  | ≥ 6 years old | 101cm ≤ HT < 140cm | std BW = 0.000127719X^3^ - 0.0414712X^2^ + 4.8575X - 184.492 |
|  |  | 140cm ≤ HT < 149cm | std BW = -0.00178766X^3^ + 0.803922X^2^ - 119.31X + 5885.03 |
|  |  | 149cm ≤ HT < 184cm | std BW = 0.000956401X^3^ - 0.462755X^2^ + 75.3058X - 4068.31 |
|  |  |  | X: HT, OI = (actual BW–std BW)/std BW × 100 (%) |

Abbreviation: BW, Body weight; HT, height; OI, obesity index; std, standard

| **Table H** Development and severity of hyperglycemia according to patient risk | | | | | | | |
| --- | --- | --- | --- | --- | --- | --- | --- |
|  |  | SR | HR | ER | T | F | Total |
| Induction phase | Grade 3 | 6 | 4 | 3 | 2 | - | 15 |
|  | Grade 4 | 0 | 1 | 1 | 0 | - | 2 |
| Re-induction phase | Grade 3 | 3 | 6 | 1 | - | 0 | 10 |
|  | Grade 4 | 0 | 1 | 0 | - | 0 | 1 |
| Maintenance phase | Grade 3 | - | 17 | 6 | 0 | 0 | 23 |
|  | Grade 4 | - | 18 | 2 | 2 | 0 | 22 |
| Other | Grade 3 | - | - | 0 | 0 | 2 | 2 |
|  | Grade 4 | - | - | 0 | 0 | 0 | 0 |
| Total |  | 9 | 47 | 13 | 4 | 2 | 75 |

ER, extremely high risk; F, induction failure; HR, high risk; SR, standard risk, T, T cell type ALL
